# Changes in the Clinical Workforce Providing Contraception and Abortion Care in the US, 2019-2021

**DOI:** 10.1001/jamanetworkopen.2022.39657

**Published:** 2022-11-01

**Authors:** Julia Strasser, Ellen Schenk, Emma Dewhurst, Candice Chen

**Affiliations:** 1 Milken Institute School of Public Health, George Washington University, Washington, District of Columbia

## Abstract

This cross-sectional study investigates changes in the workforce providing contraception and abortion services from before to during the COVID-19 pandemic.

## Introduction

The clinical workforce providing contraception and abortion care is critical for access. This workforce includes women’s health, primary care, and other clinicians.^1^ However, these clinicians face a confluence of crises that may be associated with losses in this workforce. These include COVID-19 and policies penalizing abortion care, which culminated in the 2022 *Dobbs v Jackson* decision. Using a national-level claims data set, we examined changes in the contraception and abortion workforce from 2019 through 2021.

## Methods

This cross-sectional study was approved by the George Washington University institutional review board, which waived informed consent because there was no more than minimal risk to participants. This study is reported following the STROBE reporting guideline.

We used 2019 to 2021 IQVIA preadjudicated medical claims derived from clinician offices and insurance clearinghouses, including clinician month-level counts of services for in-person contraception (ie, intrauterine devices [IUDs]), contraceptive implants, and injectable contraception), abortion (ie, misoprostol and mifepristone, dilation and curettage, dilation and evacuation, and surgical procedures), or both. Outcome variables were counts of contraception clinicians and abortion clinicians by year and service type and monthly volume changes in services. During January 2019 to December 2021, 141 837 clinicians provided at least 1 in-person contraception or abortion service; 5804 clinicians provided both services. Data analyses occurred from June 1 to July 10, 2022, using Stata statistical software version 17.0 (StataCorp).

## Results

The number of physicians decreased in 2020 for all services except medication abortion, and the number of physicians providing contraception was not reestablished to prepandemic levels in 2021. By contrast, the number of advanced practice clinicians stayed nearly identical from 2019 to 2020 and increased above prepandemic levels in 2021 (Table).

**Table.** In-Person Contraception and Abortion Clinicians by Service Type

Type of service	Clinicians, No.^a^		
	2019	2020	2021
**Physicians**			
In-person contraception			
IUD	35 778	34 582	35 448
Implant	21 589	20 599	21 064
Injectable	48 930	47 484	47 842
Total^b^	68 574	65 994	66 904
Abortion			
Medication	1100	1114	1170
Procedural	2784	2628	2878
Total^c^	3136	3020	3293
**Advanced practice clinicians**			
In-person contraception			
IUD	10 107	10 135	11 026
Implant	8409	8110	8817
Injectable	21 573	21 604	23 141
Total^b^	26 853	26 852	28 782
Abortion			
Medication	415	436	481
Procedural	49	56	66
Total^c^	427	454	500

Abbreviation: IUD, intrauterine device.

^a^ Source: IQVIA medical (Dx) and institutional (Hx) claims, 2019 to 2021, extracted on June 15, 2022.

^b^ Clinicians providing IUD, implant, and injectable services do not sum to the total number of clinicians because clinicians may have performed more than 1 type of in-person contraception service.

^c^ Clinicians providing medication and procedural abortion do not sum to the total number of clinicians because clinicians may have performed more than 1 type of abortion service.

Contraception services decreased substantially from March through May 2020 but were restored to approximately prepandemic levels later in 2020 and through 2021 (Figure). Abortion services did not distinctly decrease in early 2020. Instead, procedural abortion steadily decreased (from 8315 services in January 2019 to 5665 services in December 2021), and medication abortions steadily increased (14 347 services in January 2019 to 16 074 services in December 2021).

**Figure.**
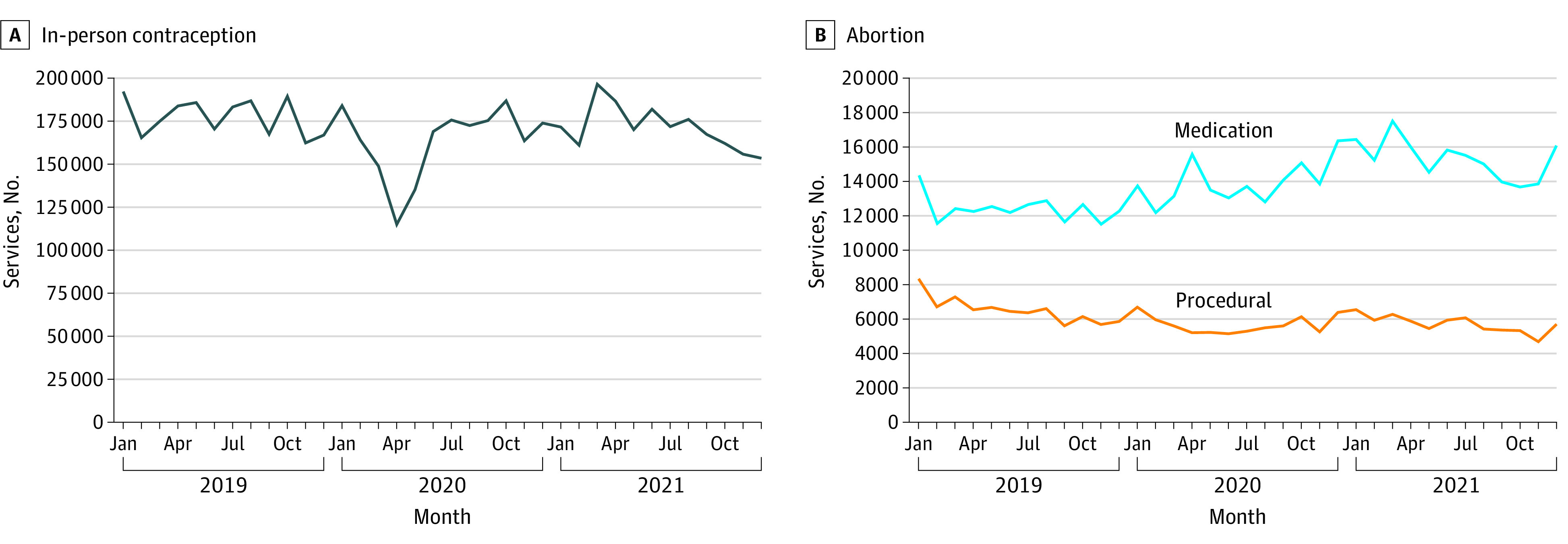
Contraception and Abortion Services Provided Source: IQVIA medical (Dx) and institutional (Hx) claims, 2019 to 2021, extracted on June 15, 2022.

## Discussion

In this cross-sectional study, we found that the overall number of clinicians providing contraception and abortion services decreased in 2020 and was restored in 2021. However, the number of physicians providing contraception services did not reach prepandemic levels, while the number of advanced practice clinicians providing contraception services continued to increase, mirroring the increasing numbers of advanced practice clinicians in primary care and other settings.^2^

COVID-19 placed incredible strain on the health workforce, with reports of practice closures and physicians leaving practice.^3,4^ While these physicians may reenter the workforce, our findings suggest there is an ongoing loss of physicians providing contraception services, which is concerning. Access requires that primary care clinicians offer the full scope of care, including family planning services. Targeted investments in women’s health and primary care clinicians and state-level expanded scope of practice policies may strengthen this segment of the workforce.

We also found that in-person contraception services decreased in the early COVID-19 period (March-May 2020) but were restored later in 2020, However, procedural abortion services decreased over the 3-year period, and medication abortion services increased, consistent with other studies.^3,5^ In 2020, many individuals reported fertility plans for fewer children or later pregnancies, suggesting that demand for contraceptive services remained even as services were unavailable.^6^

Although IQVIA medical claims contain data on 94% of physicians registered with the American Medical Association, this study provided a partial view of the contraception and abortion workforce owing to data limitations noted elsewhere.^1^ Additionally, this study did not capture outcomes associated with the 2022 Supreme Court decision overturning *Roe v. Wade,* a watershed event that will shape the provision of abortion and contraception for the foreseeable future. However, we found losses in this workforce even before 2022. It will be critical to continue to track this workforce in the coming years given that access to care intrinsically relies on the workforce.
