# Errors in Service Counts in Results and Figure

**DOI:** 10.1001/jamanetworkopen.2024.4737

**Published:** 2024-02-29

In the Original Investigation titled “Changes in the Clinical Workforce Providing Contraception and Abortion Care in the US, 2019-2021,”^1^ published November 1, 2022, services were inadvertently overcounted in Results and the Figure by including both the general procedure/visit code and the device/medication code for intrauterine device and medication abortion. The article has been corrected.

## References

[1] Strasser J, Schenk E, Dewhurst E, Chen C. Changes in the clinical workforce providing contraception and abortion care in the US, 2019-2021. JAMA Netw Open. 2022;5(11):e2239657. doi:10.1001/jamanetworkopen.2022.39657 36318211 PMC9627410

